# Defect chemistry of electrocatalysts for CO_2_ reduction

**DOI:** 10.3389/fchem.2022.1067327

**Published:** 2022-11-08

**Authors:** Hongqiang Li, Ran Li, Jiabao Niu, Kaining Gan, Xiaojun He

**Affiliations:** School of Chemistry and Chemical Engineering, Anhui Key Laboratory of Coal Clean Conversion and High Valued Utilization, Key Laboratory of Metallurgical Emission Reduction and Resources Recycling, Ministry of Education, Anhui University of Technology, Maanshan, China

**Keywords:** defects chemistry, defects engineering, CO_2_ electroreduction, carbon-based materials, metal compounds

## Abstract

Electrocatalytic CO_2_ reduction is a promising strategy for converting the greenhouse gas CO_2_ into high value-added products and achieving carbon neutrality. The rational design of electrocatalysts for CO_2_ reduction is of great significance. Defect chemistry is an important category for enhancing the intrinsic catalytic performance of electrocatalysts. Defect engineering breaks the catalytic inertia inherent in perfect structures by imparting unique electronic structures and physicochemical properties to electrocatalysts, thereby improving catalytic activity. Recently, various defective nanomaterials have been studied and show great potential in electrocatalytic CO_2_ reduction. There is an urgent need to gain insight into the effect of defects on catalytic performance. Here, we summarized the recent research advances on the design of various types of defects, including carbon-based materials (intrinsic defects, heteroatom doping and single-metal-atom sites) and metal compounds (vacancies, grain boundaries, and lattice defects). The major challenges and prospects of defect chemistry in electrocatalytic CO_2_ reduction are also proposed. This review is expected to be instructive in the development of defect engineering for CO_2_ reduction catalysts.

## Introduction

With the acceleration of industrialization, the emissions of greenhouse gas CO_2_ have also increased, which has led to serious environmental pollution and energy crisis (Yan et al., 2017). It is desirable to find a mild way to convert CO_2_ while producing high value-added fuels. Electrocatalytic reduction of CO_2_ is considered one of the ideal ways to achieve CO_2_ conversion, which involves a multi-proton coupled electron transfer process (Jin et al., 2021). However, the highly stable structure of CO_2_ makes it difficult to be activated to participate in the reduction reaction. In addition, the competitive hydrogen evolution reaction (HER) in the CO_2_ reduction reaction (CO_2_RR) process also reduces the selectivity of the desired product (Wang et al., 2018b). Therefore, there is an urgent need to find efficient catalysts to reduce the activation energy of CO_2_ and enhance the selectivity of target products (Yang C. et al., 2021).

It is well known that the performance of electrocatalysts is closely related to the charge transport capacity and intrinsic catalytic activity of the active sites (Zou and Wang, 2021). Tuning the electronic structure and optimizing the reaction interface are effective strategies to improve the catalytic performance (Lu et al., 2022). Among the various regulation strategies, defect engineering is an effective way to break the balance of local electron distribution and regulate the electron structure, which leads to unique physicochemical properties and excellent catalytic performance (Gong et al., 2009; Liu et al., 2019; Wang Q. et al., 2019; Xie et al., 2020). Obviously, the activity of CO_2_RR greatly depends on the defect chemistry of the employed catalysts (Xue et al., 2020). In general, defect engineering can be widely applied to various types of catalysts, such as intrinsic defects, heteroatom doping and single-metal-atom sites in carbon-based catalysts; and vacancies, grain boundaries and lattice defects in metal compounds (Wang et al., 2021a).

This review aims at providing a detailed discussion on defect chemistry of electrocatalysts that have been discovered and developed to date for CO_2_RR. We first discuss the effects of defects on carbon-based catalysts including intrinsic defects, heteroatom doping and single-metal-atom sites. Then, we discuss the role of defect engineering in tuning the local atomic and electronic structure of metal compounds, including vacancies, grain boundaries and lattice defects. Finally, the present challenges and future prospects are proposed to give some guidance for the optimization of defect engineering in CO_2_ reduction electrocatalysts.

## Defect engineering of carbon-based materials

### Intrinsic defects

**FIGURE 1:**
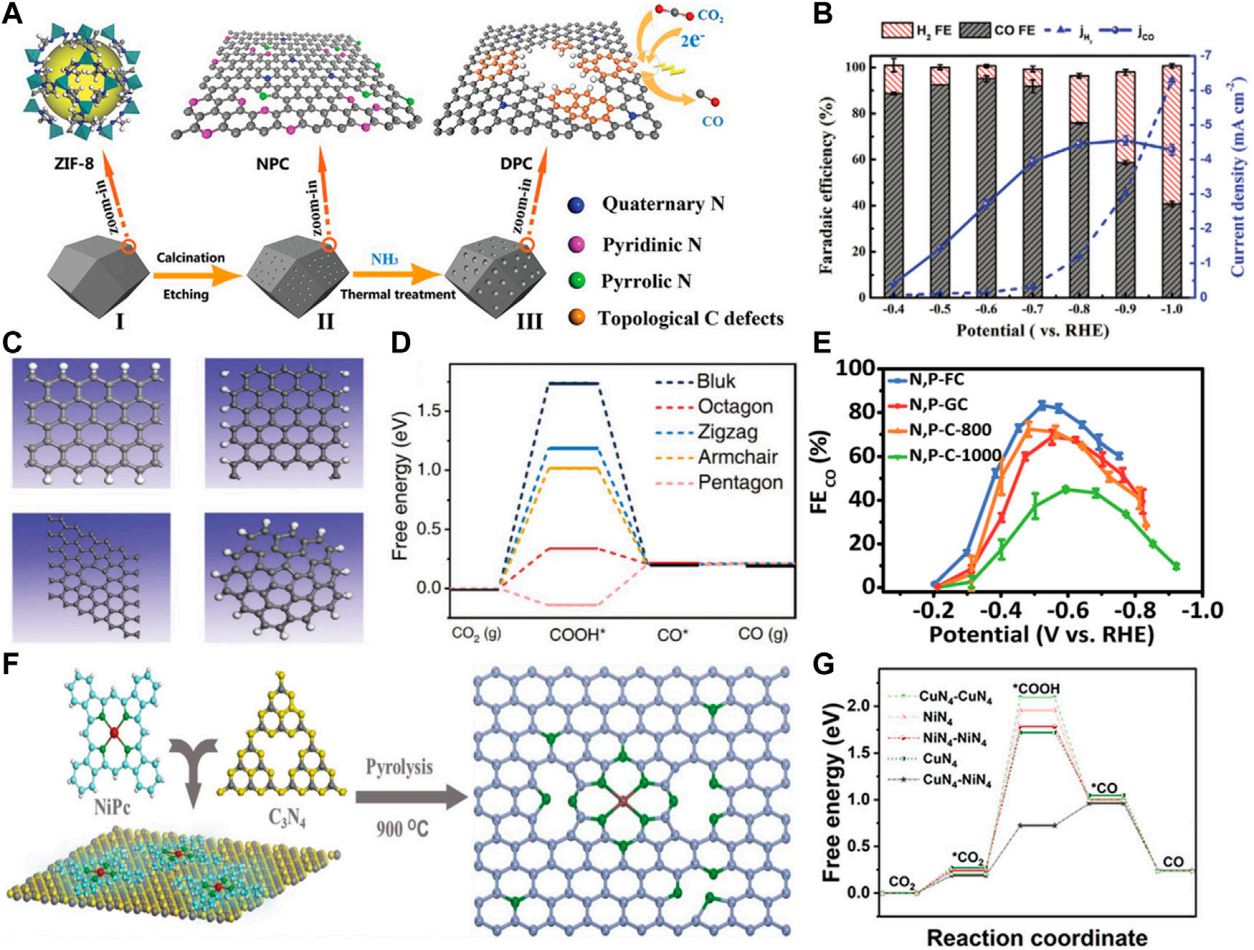
**(A)** Schematic illustration of the synthetic route of 3D topologically defected porous carbon particles. **(B)** Faradaic efficiencies of CO (gray) and H_2_ (red) and the partial current of CO on 3D topologically defected porous carbon particles under a range of applied potentials. [Reproduced from (Dong et al., 2020) with permission from the Royal Society of Chemistry]. **(C)** Defect models used for theoretical calculations: zigzag edge (upper left), armchair edge (upper right), octagonal (lower left), and pentagonal (lower right). **(D)** DFT calculations for CO_2_RR activities of different defects. [Reproduced with permission from (Wang et al., 2019)]. **(E)** FE for CO production at different applied potentials. [Reproduced from (Xue et al., 2019) with permission from the Royal Society of Chemistry]. **(F)** Schematic of the overall synthesis procedure of Ni-NG-900 catalyst. [Reproduced with permission from (Boppella et al., 2022)]. **(G)** Free energy diagram of the CO_2_RR process for CO production on various samples. [Reproduced with permission from (Hao et al., 2022)].

The intrinsic defects exist widely in carbon materials, which are formed by lattice distortion and atom deletion. The geometric configuration of intrinsic carbon defects mainly includes edges, vacancies, holes, topological defects, etc. The zigzag and armchair edges make the carbon materials full of unpaired π electrons, which can enhance the local electron density, accelerate electron transfer and lower the formation energy barriers of key intermediates. It was demonstrated that the mechanical ball milling method has the generality of producing more edge defects in the graphitic carbon skeleton (Dong et al., 2019). Meanwhile, chemical etching is an effective strategy to introduce defects on the surface of carbon materials (Li et al., 2019a). Dong et al. used NH_3_ thermal-treatment to adequately remove pyrrolic-N and pyridinic-N atoms and introduce a high density of defects (Figure 1A) (Dong et al., 2020). As a consequence, the obtained carbon materials possessed a Faradaic efficiency (FE) of 95.2% for CO production (Figure 1B), outperforming that of most metal-free CO_2_RR electrocatalysts. DFT calculations revealed that the edge pentagonal carbons with the lowest free energy dominated the active sites for CO_2_RR. This work provided a facile approach for introducing the intrinsic defects to enhance the electrocatalytic activity of carbon materials.

In addition to ammonia treatment and etching strategies, denitrification is also a common strategy for constructing topological defects. For instance, Wang et al. prepared two types of carbon materials by the high temperature removal of heteroatoms (Wang et al., 2019). They revealed that the CO_2_RR performance of carbon-based catalysts was positively correlated with the content of intrinsic carbon defects. They found that the defective porous carbons without heteroatom dopants also show impressive CO_2_RR activity. The results of synchrotron and theoretical calculations revealed that the *sp* ^*2*^ defects, such as octagonal and pentagonal, are essential for the enhancement of CO_2_RR performance (Figures 1C,D).

Daiyan et al. (2018) prepared nitrogen-removed mesoporous carbons by carrying out different heat treatments on nitrogen-doped mesoporous carbon. With the removal of the original nitrogen species, the topologically defective structure of the catalyst was formed. When applied to CO_2_ reduction, the metal-free mesoporous carbon catalyst exhibited a CO FE of 80% and a CO partial current density of −2.9 mA cm^−2^ at a low overpotential of 0.49 V. The authors demonstrated that the defect sites are the main active sites for CO_2_RR. Similarly, Chen et al. prepared carbon-based electrocatalysts with topological defects from silk cocoons for CO_2_RR (Chen et al., 2020b). The resulting electrocatalyst was able to achieve a CO FE of −89% and maintained high selectivity during the 10 days test. Theoretical calculations show that topological defects containing pentagonal carbon rings are the main active sites for accelerated electron transfer in CO_2_ reduction.

The introduction of topological defects will break the electronic symmetry of the aromatic ring, and the adjacent carbon atoms can be optimized as the active sites for CO_2_ reduction. Although the construction of intrinsic defects has been well developed, the accurate and quantitative description of certain intrinsic defects is still in its infancy. Some straightforward and simple protocols to precisely quantify the number of defects is highly demanded (Sebastian et al., 2022).

### Heteroatoms doping

By introducing a small number of other atoms into the original carbon structure, the electronegativity distribution on the surface of the original carbon structure is changed, i.e., the doping of heteroatoms. The introduction of heteroatoms will optimize the intrinsic electronic structure of carbon materials and greatly improve their electrocatalytic activity. The heteroatoms doped into carbon materials are usually nitrogen (N) (Li et al., 2018; Li et al., 2019b; Ning et al., 2022), phosphorus (P) (Chen et al., 2020a), boron (B) (Du et al., 2022), and sulfur (S) atoms (Wang et al., 2020a).

Among them, nitrogen atom doping is the most widely studied. The N doping can be divided into four configurations due to its different locations: graphitic N, pyridinic N, pyrrolic N and N oxide. In the earlier study of N doped carbon materials, Wu et al. (2015) developed N-doped carbon nanotube (NCNT) arrays as efficient electrocatalysts for CO_2_ reduction. The authors claimed that the structural nature and defect density of N in CNTs determined the catalytic activity. These studies demonstrated the vital role of various configurations of N-doped carbon materials in CO_2_RR. Wang et al., 2021b fabricated the N-doped porous carbon (NPC) from poly (aniline-co-pyrrole) copolymer with a pyridinic-N content of 2.86 wt% in the presence of salt templets. The optimized catalyst exhibited excellent CO_2_RR performance with a CO FE of 95.3%, exceeding the performance of most previous N doped carbon electrocatalysts. The experimental and theoretical calculation revealed that the pyridinic-N species contributed significantly to CO_2_RR.

Besides N, other types of heteroatoms doping/codoping have also been studied. Nakata et al. (2014) used a boron-doped diamond (BDD) electrode to produce formaldehyde under ambient conditions in seawater. This approach broke the traditional limit of the low yield of high-order products, and also suppressed the hydrogen generation. The BDD electrodes with p-type surfaces exhibited a high FE (74%) for formaldehyde, which was helpful for practical applications. The superior performance was attributed to the *sp* ^*3*^-bonded carbon of the BDD. These results have great inspiration to achieve high efficiency and low-cost CO_2_ transformation. Li et al. (2016) reported the S-doped and dual S, N-doped polymer-derived carbons could be used as catalysts for CO_2_RR. The S, N-doped carbons were obtained by treating the polymer-derived carbon with urea, which showed better catalytic activity towards CO_2_ reduction than the single S-doped carbons. Xue et al. (2019) synthesized a P and N coordinated fullerene-like carbons (N, P-FC) *via* a facile soft template pyrolysis method, which showed an impressive activity for CO production in CO_2_RR (Figure 1E).

### Single-metal-atom sites

Highly dispersed single-metal-atom sites in carbon skeleton can significantly improve the electrocatalytic performance of CO_2_RR (Wang et al., 2020b; Huang et al., 2022). Single-metal-atom catalysts have received extensive attention due to their high atomic utilization rate and excellent catalytic activity (Zhao et al., 2021). Unlike intrinsically defective or heteroatom-doped active sites, these highly dispersed metal species are generally considered to be directly available as active sites. A large body of research relating to single-metal-atom catalysts has focused on metal-nitrogen-carbon (M-N-C) materials in recent years. MNx constituted the catalytic active sites for the majority of M-N-C materials (Guo et al., 2020), which is similar to the MN_4_ sites in the macrocylic N-C complexes. For example, metalloporphyrin is a highly effective homogeneous electrocatalyst for CO_2_RR. Thus, MNx sites existing in solid carbon materials are also considered as active sites for CO_2_RR.

Research on single-metal-atom catalysts applied to CO_2_RR can be traced back to 2015. Varela et al. found that the metal-doped nitrogenated carbon, such as Fe-N-C, Mn-N-C and Fe, Mn-N-C showed high CO_2_RR performance (Varela et al., 2015). Undeniably, for M-N-C catalysts, the category of metal elements and the coordination number of the metal and nitrogen atoms are of key importance for the active sites in CO_2_RR.

The immobilization of transition metal-nitrogen-carbon complexes has been studied for decades. Various methods and techniques have been generated in this area as well. Meshitsuka et al. are among the earliest researchers who successfully immobilized CoPc and NiPc onto the surface of graphite electrodes (Meshitsuka et al., 1974). In recent years, there has been an increasing number of reports on transition M-N-C complexes as catalysts applied to CO_2_RR involving various metal centers and supports. However, it is difficult to control the number or thickness of catalyst layers for M-N-C materials prepared by absorption or immobilization methods, which may result in insufficient or too dense active centers and hinder the diffusion of CO_2_. Therefore, many researchers have been exploring other methods to prepare heterogeneous M-N-C catalysts. A metal-organic framework (MOF) is a polymer consisting of metal-organic ligands on an open framework with a coordinated, porous heterogeneous network structure.

Zhao et al. prepared Ni SAs/N-C catalyst containing Ni-N single-atom sites by ionic exchange of MOFs, showing a superior CO_2_RR performance with a CO production turnover frequency (TOF) of 5,273 h^−1^ (Zhao et al., 2017). Ye et al. (2017) synthesized Fe-N-C catalyst by surface functionalization of ZIF-8 with ammonium ferric citrate. The highly exposed Fe-N-C presented an excellent performance for CO_2_RR, achieving a CO FE of up to 93% at −0.43 V vs. RHE. Gu et al. (2019) prepared a monodisperse Fe^3+^ single-atom catalyst by controlling the pyrolytic process. The onset potential for CO production was only 80 mV. The partial current density of CO reached 94 mA cm^−2^ at an ultra-low overpotential of 340 mV. The authors revealed that the active sites were monodisperse Fe^3+^ particles coordinated with the pyrrolic N on the carbon support using the *in-situ* synchrotron X-ray absorption spectroscopy. Similarly, Boppella et al. (2022) developed soft-template assisted technology to synthesize pyrrolic N-stabilized single Ni atom electrocatalyst (Figure 1F). The results of the control experiments showed that the synergistic interaction between Ni-Nx and metal free defect sites could effectively promote CO_2_RR activity.

Furthermore, Lu et al. (2021) developed highly dispersed indium (In) atoms on an N-doped carbon (In-N-C) as a highly efficient catalyst for formic acid production. Hao et al., 2022 synthesized dual-single-atom catalysts consisting of atomically dispersed CuN_4_ and NiN_4_ bimetallic sites using electrospun carbon nanofibers. The optimal catalyst exhibited excellent CO FE of 99.6% in a wide potential range (−0.78 to −1.18 V vs. RHE) with a high turnover frequency of 2,870 h^−1^. The authors revealed that the electronegativity offset of the bimetal atoms induced the strong electron interactions, thereby accelerating the *COOH adsorption and decreasing water dissociation kinetics (Figure 1G). Wang et al. synthesized a Co-N-C catalyst dominated with isolated CoN_4_ sites (CoN_4_-CNT) (Wang et al., 2022). When employed in CO_2_RR, CoN_4_-CNT exhibited both excellent selectivity (FE_CO_ = 99.4%) and activity (*j*_CO_ = −24.8 mA cm^−2^) at a low overpotential of 0.49 V in H-type cell, and the FE_CO_ remained above 90% with increasing current density from 50 to 600 mA cm^−2^ in the flow cell.

Single-metal-atom catalysts have become a research hotspot in various catalytic fields, but there are still some doubts about the exact location of their catalytic active center. This is because there are many potential active sites in single-metal-atom catalysts, such as single-metal sites, heteroatoms, intrinsic carbon defects, etc., and it becomes more difficult to quantitatively describe these defects (Qin et al., 2019b). Therefore, more efforts are needed to accurately construct single-metal-atom catalysts with high purity and good homogeneity and to establish a linear relationship between defect density and catalytic activity.

## Defect engineering of metal compounds

The active centers of metal-based electrocatalysts are often located in defective sites as well. The reasonable introduction of appropriate defects is necessary to enhance the electrocatalytic activity. The charge distribution and intermediate adsorption of the defective active site depend on its microstructure, which is of great significance for the CO_2_RR process (Gong et al., 2019). Owning to the crucial roles of defects sites in creating new functionalities of metal compounds, atomic-scale identification and quantification is very urgent. In addition to some common evaluation techniques such as Raman spectroscopy, electron-spin resonance spectroscopy, and infrared spectroscopy, etc., the recent rise of deep machine learning may also provide a feasible route to quantify defects (Taherimakhsousi et al., 2020; Yang et al., 2021). According to the different microstructures, the defects of metal compounds can be divided into vacancies, grain boundaries, lattice defects and so on.

### Vacancies

**FIGURE 2:**
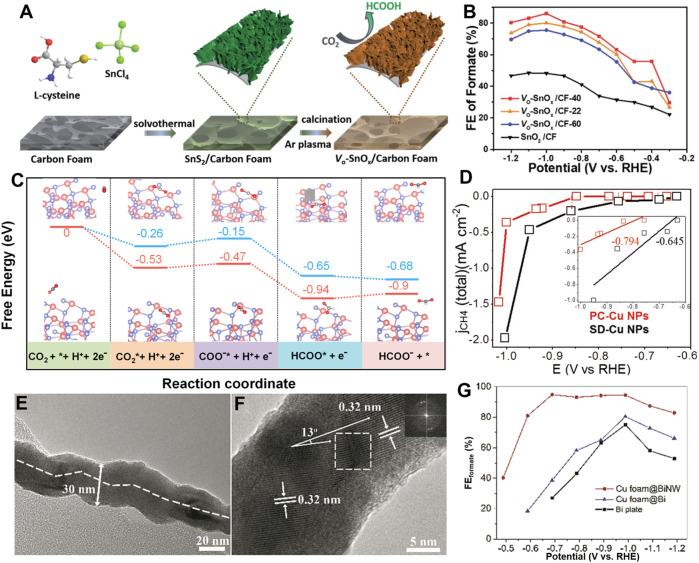
**(A)** Schematic illustration of the fabrication process of the oxygen vacancy-enriched SnO_x_ nanosheets grown on carbon foam. **(B)** FE of formate for various electrodes at different electrolytic potentials. (Reproduced from (Li et al., 2020) with permission from the Royal Society of Chemistry). **(C)** Free energy diagram for the pathways of CO_2_ conversion into formate on a perfect ZnS and V_Zn_-ZnS surface. [Reproduced with permission from (Pang et al., 2019)]. **(D)** ECSA normalized CH_4_ partial current density. [Reproduced with permission from (Choi et al., 2019)]. **(E)** TEM image of a BiNW showing a twisted structure. **(F)** HRTEM image of the BiNW showing a grain boundary with crystal lattice dislocation (inset is an FFT image of the square area). **(G)** Faradaic efficiencies of formate on Cu foam@BiNW, Cu foam@Bi and Bi plate. [Reproduced from (Zhang et al., 2019) with permission from the Royal Society of Chemistry].

Thanks to the lower formation energy, oxygen vacancies are the most common anion defects in metal oxides. The physicochemical properties of metal oxides are changed by the presence of oxygen vacancies, such as the activation of the electron-deficient carbon atoms in CO_2_, thereby promoting CO_2_RR (Cao et al., 2022; Wang et al., 2022). For example, Zeng’s group reported a facile H_2_ plasma treatment to produce oxygen vacancies-riched ZnO nanosheets (Geng et al., 2018). The obtained catalyst exhibited a CO FE of 83% while maintaining a current density of 16.1 mA cm^−2^ at −1.1 V vs. RHE. DFT calculations revealed that the charge density of ZnO near the valence band maximum increased due to the presence of oxygen vacancies, which promoted the activation of CO_2_. Li et al. (2020) synthesized oxygen vacancy-rich SnO_x_ nanosheets supported by carbon foam *via* solvothermal coupled plasma etching strategy (Figure 2A). The optimized electrode exhibited a high FE (86%) and a high partial current density (30 mA cm^−2^) for formate production (Figure 2B). The oxygen vacancies greatly increased the electrochemical surface area and boosted the adsorption and activation of CO_2_. Numerous studies have shown that the presence of oxygen vacancies can improve the efficiency of CO_2_RR by enhancing CO_2_ adsorption and facilitating its conversion to intermediates (Gao et al., 2017; Wang et al., 2018a; Han et al., 2021; Teh et al., 2021). Consequently, quantitative characterization of oxygen vacancies is thus important to the understanding and application of vacancies-rich catalysts. Lei et al. (2022) found that ZnO nanoparticles with oxygen vacancies can trigger the luminol-H_2_O_2_ system to emit strong chemiluminescence (CL), and the CL intensity is strongly dependent on the oxygen vacancies of ZnO nanoparticles. Based on this characteristic, the authors proposed one method for quantifying the oxygen defects in ZnO. In addition to oxygen vacancies, sulfur defects also play a vital role in modifying the electronic structure and reducing the reaction energy barrier of CO_2_RR (Kang et al., 2019; Cheng et al., 2020). Qin et al. prepared a catalyst of CNTs coated with CdS and found that the S-vacancies generated in the CO_2_RR process (Qin et al., 2019a). Interestingly, there was a positive correlation between the content of S vacancies and CO formation. DFT calculations indicated that the presence of S-vacancies changed the electron density of the CdS-CNTs catalyst and reduced the energy barrier of CO production.

In addition to anion vacancies, cation vacancies can also greatly affect the physicochemical properties of metal compounds (Yan et al., 2022). Thanks to their unique electron and orbital distribution, cation vacancies-rich materials also exhibit excellent electrocatalytic performance. For example, Pang et al. (2019) developed a cation vacancy-rich ZnS catalyst, achieving a high selectivity (>85%) of formate in CO_2_RR. Both experimental and calculation results have demonstrated that the surface Zn vacancies lower the barrier of CO_2_RR and suppress the HER (Figure 2C). Xia’s group reported the synthesis of ZnIn_2_S_4_ nanosheets with abundant Zn vacancies by ultrasonication strategy (Wang et al., 2021d). The obtained catalyst exhibited a partial current density of 245 mA cm^−2^ and a FE of 94% for formate production in a flow cell. The results revealed that the Zn vacancies with large electrochemically active surface area contributed to the formation of formate.

### Grain boundaries

As mentioned above, both cations and anions vacancies can further enhance the performance of CO_2_RR. In addition to vacancies, the grain boundaries in polycrystalline materials may also alter the intrinsic physical and chemical properties of the material. For example, Tang et al. (2020) synthesized a series of copper twin boundaries on polished Cu electrodes. The atoms at grain boundaries greatly contribute to methane production. Both the experiment and DFT studies confirmed the twin boundaries facilitate the conversion of absorbed CO* into CH_4_. Furthermore, Choi et al., 2019 prepared unique star decahedron Cu nanoparticles (SD-Cu NPs) with twin boundaries and multiple stacking faults, exhibiting a low onset potential for methane formation in CO_2_RR. The DFT calculations revealed that the twin boundaries significantly reduced the formation energy of *CHO, thereby boosting the formation of CH_4_ at low overpotentials (Figure 2D). Wang et al., 2021c prepared carbon sustained SnO_2_-Bi_2_O_3_ hollow nanofibers as Janus catalyst for formate production. The common boundary of the two components synergistically promotes intrinsic activity. All these suggest that grain boundaries may promote the charge density distribution or change the local electronic environment, thus improving the electrocatalytic performance of metal-based catalysts.

### Lattice defects

Lattice defects, such as lattice dislocations, expansion and distortion, may significantly alter the physicochemical properties of the metal compounds. The unique electronic structure and enhanced electrical conductivity provided by lattice defects help accelerate charge transport during the CO_2_RR, thereby increasing catalytic activity. For instance, Zhang’s group synthesized *in situ* AgCo surface alloy electrocatalysts by cold H_2_-plasma activation method (Zhang et al., 2020). The as-prepared alloy exhibited high FE of ethanol (72.3%) when used as a CO_2_RR electrocatalyst. The experimental and DFT results revealed that the distortion of the Ag lattice reduced the energy barrier for the intermediate formation and enhanced the C-C coupling. Zhou et al., 2020 used a supercritical CO_2_-assisted strategy to fabricate intralayer [Bi_2_O_2_]^2+^ structural distortion in BiOCl. Thanks to the abundant intralayer [Bi_2_O_2_]^2+^ structural distortions in thin nanoplate of BiOCl, the catalyst showed a high selectivity of formate (92%) production in a broad potential range from −0.6 to −0.9 V vs. RHE. This work provides a new route to synthesize two-dimensional materials with crystal distortions for electrocatalytic applications.

Zhang et al. (2019) developed a facile *in situ* electrochemical transformation strategy to synthesize copper foam supported lattice-dislocated Bi nanowires (Cu foam@BiNW). The obtained Cu foam@BiNW showed an enhanced CO_2_RR performance with a FE for formate of 95% at −0.69 V vs. RHE (Figures 2E–G). The high CO_2_RR activity is attributed to the lattice dislocations on the twisted Bi nanowires and the porous structure. They also synthesized a defect-rich Bi catalyst from Bi_2_S_3_ through a one-pot hydrothermal reaction (Zhang et al., 2018). When applied to CO_2_RR, the as-prepared catalyst exhibited the maximum FE of 84% for formate production at an overpotential of 670 mV. The authors revealed that the lattice defects associated with the Bi_2_S_3_-derived Bi might have a positive effect on CO_2_RR.

## Conclusion and perspectives

The application of defective catalysts in electrocatalytic CO_2_ reduction has become a new research hotspot and has attracted extensive attention in recent years. Defect engineering provides an efficient strategy to tune the surface physicochemical properties of electrocatalysts and leads to a substantial improvement in catalytic performance. In this review, we have briefly summarized recent research progress in defect engineering of electrocatalysts applied to CO_2_ reduction. Various strategies for adjusting and modifying the surface defects of catalysts are summarized, including intrinsic defects, heteroatom doping, single-metal-atom sites, vacancies, grain boundaries and lattice defects. These defects can adjust the electronic structure of the active center of the electrocatalyst, thus affecting the adsorption, activation and conversion of CO_2_. Although significant progress has been made in existing studies of catalytic defects, there are still a series of difficulties and challenges remains to be considered.

1 Precise construction of defective sites. It is difficult to precisely introduce a specific defect into the skeleton or lattice of a material. In fact, it is difficult to synthesize catalysts with only one type of defect. For example, there are often multiple types of intrinsic defects in carbon materials, such as edge sites, vacancies, topological defects, and possibly accompanying heteroatoms, etc. Similarly, in metal-based defective materials, lattice dislocations, expansion and distortion are often present simultaneously. In order to determine the specific effects of defect species on electrocatalytic reactions, more controllable and accurate construction methods should be developed. In particular, attempts should be advocated to develop new methods for the large-scale construction of defective materials that can control defect types and density.
2 Advanced characterization techniques to identify defects. Defects often bring unexpected effects. For example, the electronic structure and surface state at the defect site show different properties from the matrix structure. Thus, it becomes more important to characterize them in depth and detail. Although there are many effective techniques and methods for the qualitative characterization of defective sites, the quantitative description of defective sites has always faced some challenges. Therefore, it is highly desirable to accurately characterize defects qualitatively and quantitatively, which will help us in defect site design and predicting the effect of defects on catalytic performance.
3 Stability of defective sites during electrocatalytic reactions. A non-negligible issue during catalytic reactions is the evolution of defective active centers in a complex electrochemical environment. Namely, the stability of the defective sites is an important parameter for practical applications. Changes in defects during the reaction process can significantly increase the complexity of the study, as structural changes often lead to changes in the reaction mechanism. Many unstable defective structures tend to undergo irreversible reconstruction or phase transitions when driven by electric fields, which requires *in situ* or operando observation of the defect sites during reaction process.
4 Mechanism of defect-sites catalyzed CO_2_ reduction. Although significant advances have been made in defect engineering for CO_2_RR, the mechanistic study of defective sites on the CO_2_ reduction process is still in its initial stage. The adsorption and transformation behavior of CO_2_ molecules and intermediates at defective sites, and the C-C coupling mechanism of multi-carbon products need to be further clarified. Advanced theoretical simulations and characterizations can help us to understand the active sites and dissect the reaction process, and to explore the structure-activity relationships and reaction mechanisms to a certain extent. Considering the disparity between theoretical calculations and experimental studies, the sophisticated construction of simulation models may be a key step in breaking the gap between theory and reality, and thus deserves more attention.

In summary, we have described the development of defect engineering in the design of CO_2_RR electrocatalysts. It is obvious that exploring the nature of defects is important for understanding the defective active center and its reaction mechanism in catalyzing CO_2_ reduction. At this urgent time of increasing carbon emissions, efforts are needed to develop promising defect-rich catalysts that can be prepared on a large scale for high value-added conversion and utilization of CO_2_. Although there are still many shortcomings in the current CO_2_ reduction system, we believe that the combination of precisely controlled construction methods, advanced characterization techniques, scientific experimental studies and rigorous model simulations will further accelerate the commercial application of defective electrocatalysts for CO_2_RR.
